# Clinical utility of consecutive volume scanning dual‐energy CT in differentiating hemorrhage from contrast staining in ischemic stroke patients

**DOI:** 10.1002/acm2.70209

**Published:** 2025-08-21

**Authors:** Aroon Pressram, Reordan DeJesus, Tara Massini, Anna Y. Khanna, Manuel Arreola, Izabella L. Barreto

**Affiliations:** ^1^ Department of Department of Radiology University of Florida Gainesville Florida USA; ^2^ Department of Vascular Neurosurgery University of Florida Gainesville Florida USA

**Keywords:** dual‐energy CT, iodine map, ischemic strokes, non‐contrast CT, stroke imaging, virtual monochromatic images, virtual non‐contrast images

## Abstract

**Background and Purpose:**

Accurate differentiation between hemorrhage and iodinated contrast staining is critical for managing ischemic stroke patients following revascularization. While dual‐energy CT (DECT) has shown promise in this context, studies have predominantly focused on dual‐source or fast‐kV switching systems. This study evaluates the diagnostic accuracy of a sequential axial scanning DECT system for assessing hemorrhagic transformations of ischemic stroke patients after having received thrombolytic therapy and/or endovascular procedures using MRI as the gold standard.

**Materials and Methods:**

A retrospective cohort of 97 ischemic stroke patients underwent DECT imaging within 24 h post‐revascularization, followed by MRI within 48 h. Patient hemorrhage types were classified based on the Heidelberg classification using MRI as a ground truth. DECT performance was assessed by calculating sensitivity, specificity, and predictive values for hemorrhage classes.

**Results:**

Of the 97 DECT examinations, 25 (25.8%) showed a hyper density in the DECT images compared to 31 (32.0%) hyper densities identified by MRI. DECT achieved 100% sensitivity for larger hemorrhages that impact patient management (class 3) but lower sensitivity (61.0%) for smaller hemorrhages (class 1) with no false positives (100% specificity).

**Conclusion:**

Sequential axial scanning DECT offers a reliable and accessible alternative to MRI for detecting clinically significant hemorrhages in acute stroke settings. Its ability to differentiate hemorrhage from contrast staining in a single session supports its integration into routine clinical workflows, enhancing timely decision‐making and improving patient care.

## 1 INTRODUCTION

Stroke is defined as the sudden loss of neurologic function.^1^ It is classified as either an ischemic stroke, due to thrombosis, embolism, or systemic hypoperfusion, or as a hemorrhagic stroke, resulting from intracerebral hemorrhage or subarachnoid hemorrhage.^2^ Ischemic stroke is the most common type of stroke, representing approximately 87% of all strokes, and occurs due to the occlusion of an artery or vein that deprives brain cells of oxygen and glucose.^3^ Advancements in the treatment of ischemic stroke have led to reduced mortality, with stroke dropping from the third to the fifth leading cause of death in the United States since the year 2000.^4^ Treatment involves recanalizing the occluded vessel early enough to restore reperfusion and minimize irreversible brain damage. This reperfusion therapy is achieved through intravenous administration of thrombolytic agents and/or mechanical thrombectomy using a stent‐retriever device.^5^

Neurological imaging plays a key role in stroke diagnosis, treatment planning, and post‐intervention monitoring. Patients that present at the hospital with stroke symptoms are typically examined with an initial non‐contrast computed tomography (NCCT) to differentiate ischemic from hemorrhagic strokes.^6^, ^7^ In the absence of hemorrhage, patients with ischemia may receive thrombolytic therapy.^8^ If CT angiography (CTA) with iodinated contrast is available, it can also be used to examine cerebral vasculature, localize vessel occlusion sites, and evaluate collateral circulation, often supplemented with concurrent perfusion analysis.^6^, ^7^

Patients with vessel occlusion are likely to receive treatment to remove the clot and achieve reperfusion in the brain. However, such interventions carry an increased risk of causing subsequent hemorrhagic transformation (HT) due to a disruption of the blood brain barrier (BBB).^9^, ^10^ Thus, patients may be examined with another follow‐up NCCT after their intervention to evaluate for HT.^11^ Patients who received iodinated contrast during their initial diagnostic CTA or mechanical thrombectomy intervention may exhibit residual iodinated contrast in the brain parenchyma, often referred to as contrast staining.^12^ On NCCT, both hemorrhage and contrast staining appear hyperdense, making it difficult to reliably distinguish between the two. If hemorrhage is detected, physicians may need to adjust or discontinue antithrombotic therapy to prevent further bleeding.^13^ Conversely, if contrast staining is misinterpreted as hemorrhage, unnecessary cessation of therapy could increase the risk of thrombotic complications. Traditionally, this differentiation has relied on MRI with susceptibility‐weighted images (SWI), where hemorrhage causes susceptibility artifact and iodinated contrast does not. The differentiation may also rely on a follow‐up NCCT at a later time, where contrast material resolves over time and hemorrhage remains stable or increases.^14^, ^15^ However, critical interventions may be delayed when follow‐up imaging is performed with MRI, due to its limited availability, longer scan times, or contraindications, or with NCCT, with the need to wait for the contrast and hemorrhage to evolve. Dual energy CT (DECT) scanners have become increasingly integrated into clinical practice and are utilized for many clinical applications. If DECT can provide a reliable alternative for imaging stroke patients, it could assist in making treatment decisions more efficiently. By acquiring images with two different energy spectra, DECT enables material decomposition, generating iodine map and virtual non‐contrast (VNC) images.^16^ These capabilities allow for differentiation between contrast staining and hemorrhage.

Previous studies have shown that DECT improves accuracy and diagnostic confidence in differentiating intracranial hemorrhage from contrast extravasation in stroke patients following revascularization.^12^, ^17^, ^18^, ^19^, ^20^, ^21^, ^22^, ^23^, ^24^, ^25^ This study aimed to build on previous work by addressing key gaps in the current literature. First, some studies compared DECT to the patient's follow‐up imaging performed with either MRI or NCCT, whichever was available,^12^, ^17^, ^18^, ^22^, ^26^, ^27^ and others only compared DECT to follow‐up NCCT.^19^, ^20^, ^21^, ^23^, ^24^, ^25^, ^28^ Our study exclusively compared DECT to follow‐up imaging with SWI‐MRI, the gold standard for detecting intracranial hemorrhage, allowing for a more robust evaluation of DECT performance. Secondly, few have assessed the ability of DECT in detecting and classifying different hemorrhagic classes and subtypes. Since CT may be less sensitive to small or subtle hemorrhages, this study performed a comprehensive assessment of DECT's performance for different hemorrhage types, according to the Heidelberg bleeding classification, in order to better understand its potential limitations and clinical implications. Third, while researchers have investigated dual‐source DECT,^12^, ^18^, ^19^, ^20^, ^21^, ^22^, ^23^, ^24^, ^25^ tin‐filtered DECT,^17^ and fast‐kV switching DECT,^26^ their findings may not be generalizable across different DECT technologies. To our knowledge, no studies have evaluated DECT in stroke imaging using sequential axial systems, such as the Aquilion ONE Genesis Edition (Canon Medical Systems, Otawara, Japan). This system acquires two sequential wide axial scans with a short time interval in between scans, performs projection‐based material decomposition, and allows utilization of automatic exposure control (AEC) system to select optimal tube current values based on the anatomy being imaged, matching the image noise between low‐ and high‐energy acquisitions.^29^ However, given the circularity of the head, AEC was not used in this study. With the growing adoption of DECT systems in clinical settings, understanding the diagnostic performance of this specific system is essential for clinical implementation.

This study evaluates the diagnostic performance of a sequential axial scanning DECT system in differentiating hemorrhagic transformation from iodinated contrast staining and in grading hemorrhages based on the Heidelberg bleeding classification, using MRI as the reference standard. By focusing on the clinically relevant outcomes, our findings aim to establish this DECT system as a practical and effective imaging tool in acute stroke management.

## 2 MATERIALS AND METHODS

### 2.1 Study design and patient population

DECT was incorporated into the clinical workflow to perform follow‐up brain imaging within 24 h after revascularization therapy in ischemic stroke patients. If DECT images revealed any hyperdense findings, or if the patient's mental status deteriorated, a brain MRI was also considered for further evaluation. Patients with contraindications such as claustrophobia, non‐removable metallic devices, or physically unstable patients did not receive a follow‐up MRI.

Institutional review board approval was obtained to collect retrospective study data. A radiology analytical software (mPower Clinical Analytics, Nuance, Burlington, MA) was queried for all patients examined with a brain DECT protocol at our hospital over a 4‐year period. Clinical indications in radiological reports and medical records were reviewed in the hospital's electronic health records system (Epic Electronic Health Record, Epic Systems Corp, Madison, Wisconsin). The following parameters were recorded: age, sex, type of recanalization therapy (intravascular thrombolysis, mechanical thrombectomy, or both), type of imaging examinations since arrival to hospital with suspected stroke (CT, CTA, DECT, MRI), time between DECT and MRI, and radiological impression. Interventions were determined by the stroke team based on clinical assessments made by neuroradiologists.

Patients were excluded from the study if they were younger than 18 years old, if the brain DECT was performed for clinical indications other than ischemic stroke, or if they lacked a follow‐up MRI within 48 h after the DECT. In cases where patients had multiple DECT scans, the DECT examination performed closest to the follow‐up MRI scan was evaluated.

### 2.2 Radiological examination

All DECT examinations were performed on a 320‐detector row CT scanner (Aquilion ONE Genesis Edition, Canon Medical Systems, Otawara, Japan) by acquiring two sequential 16‐cm wide axial scans using the scan parameters outlined in Table 1. While the DECT's AEC system can automatically select optimal tube current (mA) values to achieve equivalent image noise across high‐ and low‐energy acquisitions based on patient anatomy, it was not utilized for head protocols due to the relatively consistent head size among patients, as is standard in our conventional adult head CT imaging. For our brain DECT protocol, the scanner determined an optimal mA separation to maintain equivalent image noise between the two energy acquisitions, but these values remained constant across all patients. Using the scan projection data on the scanner's workstation, three material decomposition was performed directly on the projection data to reconstruct the iodine map (IM) image series.^30^ Our scanner's three material decomposition for our stroke protocol is characterized by a set of three basis materials defined by the vendor: iodine, water, and calcium.^29^, ^31^ By subtracting the iodine signal from 66‐keV virtual monoenergetic images, virtual non‐contrast (VNC) images were generated at the system console. The iodinated contrast appears hyperdense in the IM series, whereas only bone and hemorrhagic blood appear hyperdense in the VNC images. All images were reconstructed as 1.0‐mm thick axial slices with an increment of 1.0 mm using iterative reconstruction with standard strength (AIDR‐3D, Canon Medical Systems, Otawara, Japan). After postprocessing, all images were transferred to the hospital's picture archiving and communication system (Visage Imaging Inc, Pro Medicus Limited, Victoria, Australia).

**TABLE 1:** DECT scanning parameters for ischemic stroke patients < 24 h post‐intervention.

Scan parameter	Technique
Scan mode	Axial
kVp (low, high)	80, 135
Scan FOV (cm)	22
Tube current (mA)	Fixed: 290, 50
Rotation time (s)	0.75
Detector row configuration	160 × 0.5 mm
Scan range (cm)	16
Reconstruction kernel	FC64
Reconstruction type	Iterative reconstruction (AIDR‐3D)
CTDI_vol_ (mGy)	54.6
DLP (mGy‐cm)	873.6

Follow‐up brain MRI examinations include T1‐weighted images, T2‐weighted images, susceptibility‐weighted images (SWI), fluid‐attenuated inversion recovery images (FLAIR), and diffusion‐weighted images (DWI), performed using 1.5 T (Skyra, Siemens Healthineers USA, Malvern, PA), 1.5 T (Aera, Siemens Healthineers USA, Malvern, PA), or 3 T (Prisma, Siemens Healthineers USA, Malvern, PA) MRI scanners. DWI images allow for evaluation of the extent of infarction, T1‐TSE and T1‐MPRAGE images give better visualization of subacute blood and laminar necrosis, SWI images are sensitive in visualizing hemorrhages and microbleeds, T2‐TSE images provide useful information on brain infarction by detecting loss of arterial flow in regions of the brain, and both T2‐TSE and FLAIR images are used to evaluate extent of edema as well as the presence of additional older infarctions in the brain.^32^, ^33^

### 2.3 Image analysis

All DECT examinations were independently interpreted by seven fellowship‐trained neuro‐radiologists. The radiological impression reports were reviewed from the electronic health records system (Epic Electronic Health Record, Epic Systems Corp, Madison, Wisconsin) to identify examinations with hyper densities consistent with acute hemorrhage, contrast staining, both, or neither. Patient images were then independently reviewed by two neuroradiologists (TM, 12 years of experience; RD, 13 years of experience) to confirm the clinical diagnosis and classify hemorrhages based on the Heidelberg classification system. Briefly, classes 1 and 2 reflect smaller and larger hemorrhages within the area of infarcted tissue, respectively, while class 3 reflects blood outside of the area of infarction, including those in extra‐axial spaces.^34^ Subtypes 1A, 1B, 1C, PH2, 3A, 3B, 3C, and 3D further divide the classes based on the description provided in Table 2. Cases in which there were disagreements in hemorrhage classification were later presented to both radiologists and consensus agreement was met. In cases where patients exhibited multiple hemorrhages of different classes, the higher‐class hemorrhage was recorded, as these classes would impact the patient's course of treatment. The corresponding MRI studies were reviewed by the same neuroradiologists to identify the presence or absence of hemorrhage, as determined by signal loss and blooming on the SWI MRI series. In cases with hyperdense regions on the DECT IM series, the absence of signal abnormality on SWI MRI indirectly confirmed presence of iodinated contrast staining.^35^

**TABLE 2:** Heidelberg table for classifying bleeds.

Class	Bleed type	Description
1	1A	Scattered small petechiae, no mass effect
	1B	Confluent petechiae, no mass effect
	1C	Hematoma within infarcted tissue, occupying <30%, no substantive mass effect
2	PH2	Hematoma occupying ≥30% of the infarcted tissue, with obvious mass effect (PH2)
3	3A	Parenchymal hematoma remote from infarcted brain tissue
	3B	Intraventricular hemorrhage
	3C	Subarachnoid hemorrhage
	3D	Subdural hemorrhage

### 2.4 Statistical analysis

Statistical analysis was conducted using software (MedCalc, Ostend, West‐Vlaanderen, Belgium) to calculate sensitivity, specificity, positive predictive value (PPV), and negative predictive value (NPV) with 95% confidence intervals (CI). These metrics were stratified by hemorrhage class to assess diagnostic performance of DECT while using MRI as the reference standard.

## 3 RESULTS

Of 418 patients examined with a brain DECT protocol, 97 met the inclusion criteria (53 females, 44 males, mean age 66 years, range 24–95 years). Interventions included thrombolytic therapy (*n* = 55, 56.7%), mechanical thrombectomy (*n* = 20, 20.6%), and a combination of both therapies (*n* = 22, 22.7%). In addition, 2 (2.1%) patients failed reperfusion on a primary mechanical thrombectomy and underwent emergent secondary mechanical thrombectomy. The average time interval between DECT and follow‐up MRI for patients that met the inclusion criteria was 18 h and 15 min.

**FIGURE 1:**
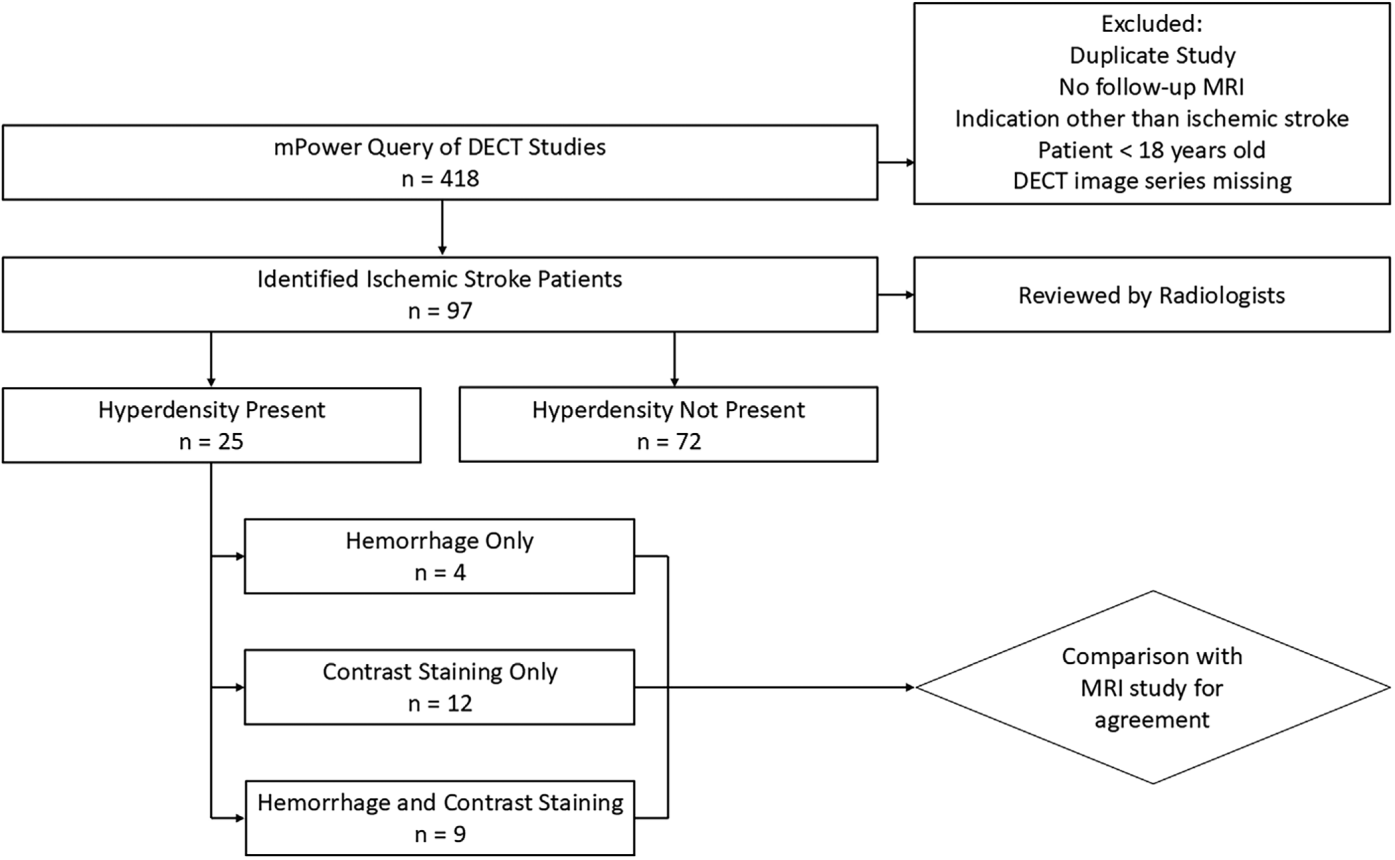
Flow diagram illustrating patient selection criteria and review.

Figure 1 shows selection criteria and study outline. Table 3 lists MRI and DECT detection of hemorrhage subtypes and contrast extravasation. Of the 97 studies, no hyperdense signal was present in 66 MRI and 72 DECT studies. Of the 31 MRIs that had hyperdense signal, all were hemorrhages, of which 25 were classes 1 and 6 were class 3. MRI is unable to detect contrast staining, although it can confirm contrast staining detected in the DECT VNC based on a lack of signal in the SWI MRI. Of the 25 DECT exams that had hyperdense signal, the IM and VNC series indicated four were hemorrhage only, nine were both hemorrhage and contrast staining, and 12 were contrast staining only. DECT agreed with MRI in 79 of 97 cases (81.4%), including 62 cases with no hyperdensities, 6/6 (100%) cases with class 3 hemorrhages, 7/25 (28%) cases with class 1 hemorrhages. Of the 12 cases that DECT called iodine staining alone, MRI demonstrated class 1 hemorrhages in eight of these, suggesting only four cases were only demonstrating iodine staining. Cases that were not in agreement included 18 cases of class 1 hemorrhages detected by MRI but not DECT. Of these 18, DECT called eight to be contrast staining only and 10 to be negative for any hyper densities.

**TABLE 3:** Number of patients with hyper densities, contrast extravasation, and hemorrhages with subtypes classified using the Heidelberg classification system.

	MRI	DECT
Total cases	97	97
No hyperdensity present	66	72
Any hyperdensity present *	31	25
Only hemorrhage present	31	4
Both hemorrhage and contrast present	N/A ^**^	9
Only contrast present	N/A ^**^	12

Hemorrhage type	Subtype		Total	Total
	1A	9	25	7 (3 with hemorrhage only, 4 also with contrast staining)
	1B	12		
	1C	4		
	PH2	0	0	0
	3A	1	6	6 (1 with hemorrhage only, 5 also with contrast staining)
	3B	2		
	3C	3		
	3D	0		

*Note*: ^*^Any hyper density present in magnetic resonance imaging (MRI) susceptibility weighted imaging (SWI) indicates hemorrhage only, in dual energy computed tomography (DECT) indicates hemorrhage and/or contrast. ^**^MRI cannot detect contrast staining.

Table 4 lists the sensitivity, specificity, PPV, and NPV of different classes of hemorrhages for DECT compared to MRI. For class 1 hemorrhages, DECT demonstrated a sensitivity of 61.0% (95% CI: 44.5–75.8), specificity of 100% (95% CI: 94.5–100), PPV of 100% (95% CI: 86.3–100), and NPV of 80.5% (95% CI: 73.8–85.8). For detection of class 3 hemorrhages, DECT achieved a sensitivity and specificity of 100% (95% CI: 54.1–100) and 100% (95% CI: 94.6–100), respectively, with a PPV and NPV of 100% (95% CI: 100%–100%) and 100% (95% CI: 94.6–100) respectively. Specificity was 100% for all hemorrhage types due to a lack of false positive findings.

**TABLE 4:** Statistical results of DECT compared to MRI.

	Class 1	Class 3
Sensitivity	61.0 (44.5–75.8)	100.0 (54.1–100.0)
Specificity	100.0 (94.6–100)	100.0 (94.6–100)
NPV	80.5 (73.8–85.8)	100.0 (94.6–100)
PPV	100.0	100.0

*Note*: Parentheses represent 95% confidence interval.

**FIGURE 2:**
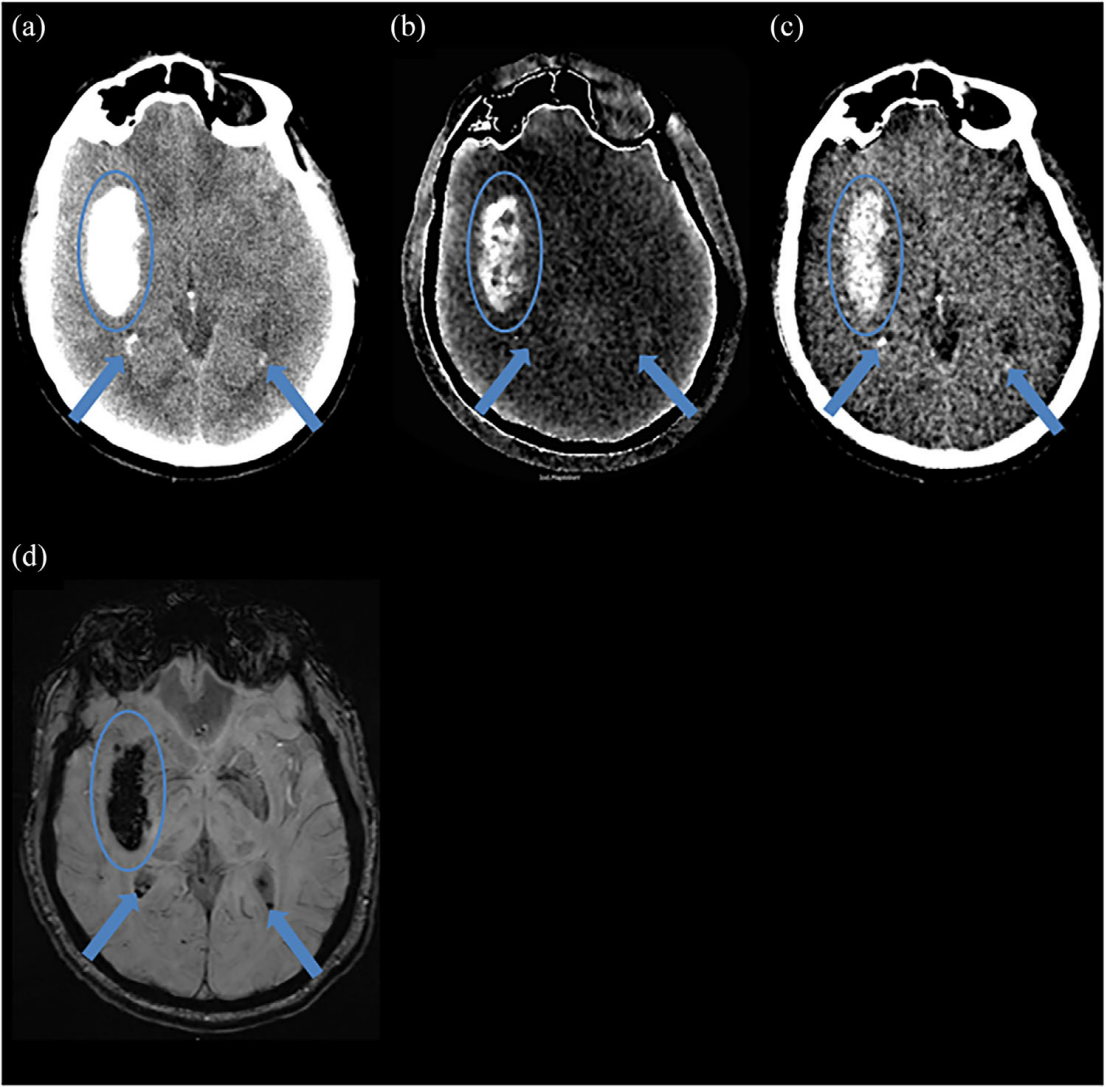
Example of a class 3 hemorrhage agreement. Fifty‐three‐year‐old male with small intraventricular hemorrhage (denoted by arrows) and large intraparenchymal hemorrhage (denoted by circles) after receiving mechanical thrombectomy. Magnetic resonance imaging (MRI) was obtained approximately 13 h after dual energy computed tomography (DECT). (a) 66 keV image shows areas of hyper densities denoted by the arrow and the circle, (b) Iodine map displays iodine as a hyperdense structure denoted by the circle suggesting the presence of contrast staining (c) Virtual non‐contrast image confirms presence of coexistent blood by the circle and the arrow, (d) MRI susceptibility weighted imaging (SWI) showing hemorrhages by the circle and the arrow.

**FIGURE 3:**
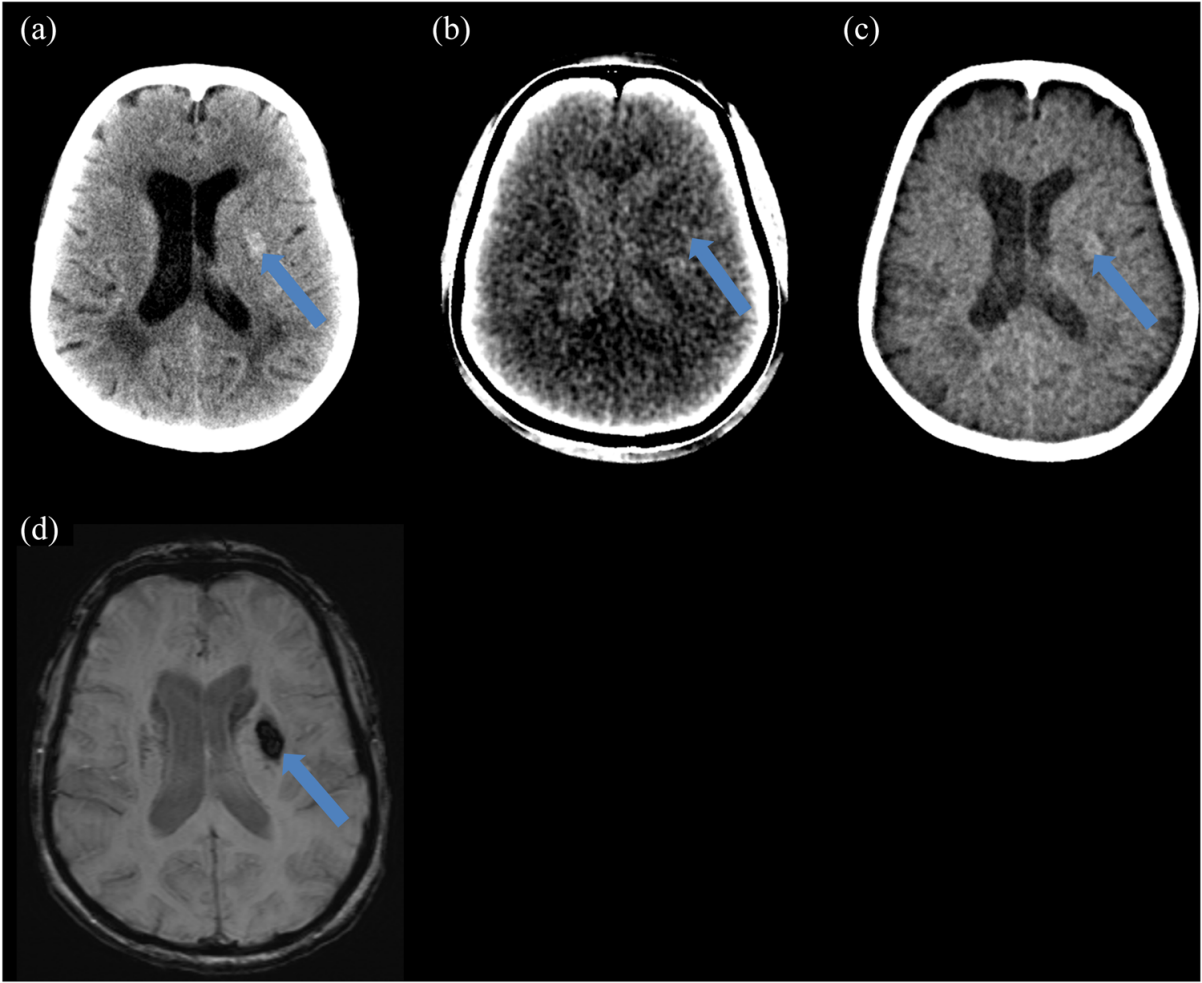
Example of a class 1 hemorrhage agreement. Female 71 years old with a confluent petechial hemorrhage (denoted by arrows) after mechanical thrombectomy. Magnetic resonance imaging (MRI) was obtained approximately 13 h and 27 min after dual energy computed tomography (DECT). (a) 66 keV image shows both contrast extravasation and hemorrhage (arrow), (b) iodine map is negative for contrast extravasation due to a lack of hyper density by the area indicated by the arrow, (c) virtual non‐contrast image showing the hemorrhage as a faint hyper density (arrow), (d) MRI susceptibility weighted imaging (SWI) showing hemorrhage as a hypodense structure (arrow).

Figure 2 demonstrates an example of agreement between MRI and DECT of a class 3 hemorrhage, showing both hemorrhagic transformation and contrast accumulation within the area of the infarct and small volume intraventricular blood. Figure 3 demonstrates agreement between MRI and DECT in detecting a class 1 hemorrhage in the brain parenchyma.

**FIGURE 4:**
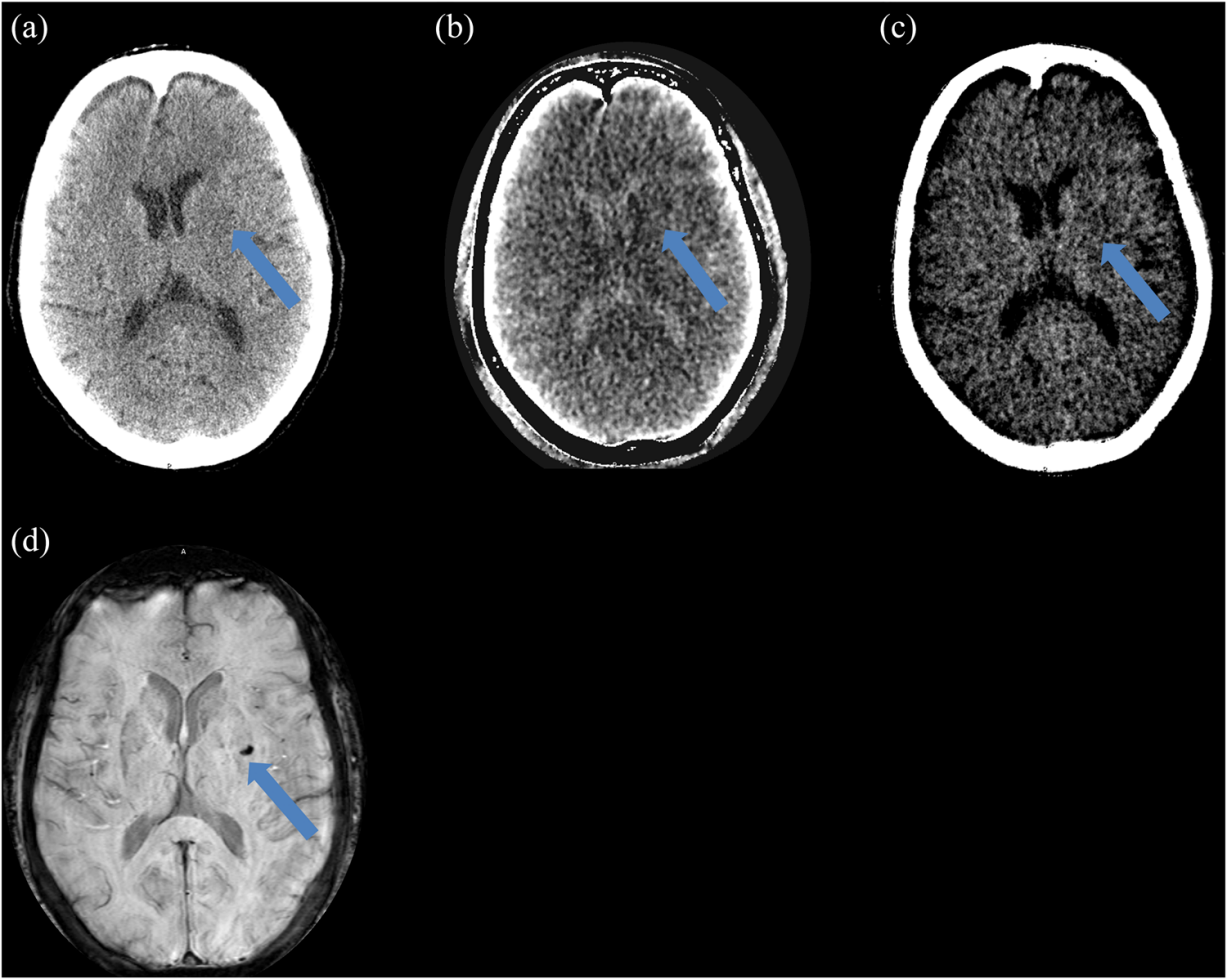
Example of a class 1 hemorrhage disagreement. Male 60 years old with a hemorrhage (denoted by arrows) after tissue plasminogen activator (tPA) administration. Magnetic resonance imaging (MRI) was obtained approximately 18 h after dual energy computed tomography (DECT). (a) 66 keV image is negative due to a lack of hyper density by the area indicated by the arrow, (b) Iodine map is negative for contrast extravasation due to a lack of hyper density by the area indicated by the arrow (c) Virtual non‐contrast image is negative for bleeds due to a lack of hyper density by the area indicated by the arrow, (d) MRI susceptibility weighted imaging (SWI) shows class 1 hemorrhage as a hypodense structure by the arrow.

Figure 4 demonstrates a case of discordance between DECT and MRI in the detection of a class 1 hemorrhage. The distribution of hyper densities on the monochromatic 66 keV images, iodine map series, and VNC images, along with the follow‐up MRI SWI sequence, are included for comparison.

## 4 DISCUSSION

This study assessed the diagnostic accuracy of a sequential axial scanning DECT system for detecting and grading hemorrhagic transformations in ischemic stroke patients, using MRI as the gold standard. Our study addresses the gap in literature by exclusively comparing against follow‐up imaging with SWI MRI, which is more sensitive to small hemorrhages, while also providing a more comprehensive evaluation of DECT's diagnostic performance by leveraging the Heidelberg classification system, offering novel insights into its detection capabilities across hemorrhage classes. Our results demonstrate that DECT provides excellent sensitivity and specificity for detecting larger, clinically significant hemorrhages (class 3), with 100% agreement with MRI. However, DECT's sensitivity for smaller hemorrhages (class 1) was lower, leading to potential under‐detection of microbleeds.

Accurately distinguishing hemorrhages from contrast staining is crucial, as it may prompt the cessation of medically‐induced coagulopathy, including the discontinuation of thrombolytics, anticoagulants, and/or antiplatelet medications.^13^ It may also involve corrective measures to revert the coagulopathy with agents such as Prothrombin complex concentrate (PCC), Vitamin K, cryoprecipitate, or fresh frozen plasma, leading to a potentially better clinical outcome.^36^

Smaller hemorrhages do not have established targeted interventions^37^, ^38^ or agreement on their clinical significance, where some studies suggest that small brain bleeds may indicate reperfusion of viable brain tissue,^10^ and others raise concerns about potential progression to larger hemorrhages.^39^ Despite these differing interpretations, routine management protocols do not recommend intervention based solely on small hemorrhages detected on imaging, and follow‐up imaging is typically performed only if patients exhibit neurological deterioration.^37^, ^38^ Thus, the lower sensitivity of DECT for class 1 hemorrhages is unlikely to impact patient management. Overall, our findings highlight the clinical relevance for radiologists, showing that while small class 1 hemorrhages may be visible on MRI but not DECT, larger hemorrhages should be detectable by both. This supports DECT as a reliable tool for identifying hemorrhages requiring clinical intervention, reinforcing its role as a practical alternative to MRI.

Our study reported a specificity of 100%, with no false positives identified. This performance is similar or superior to other studies who compared DECT with follow‐up imaging using only NCCT (range 67.7%–100%)^20^, ^24^, ^25^, ^28^ or with a combination of either MRI or NCCT (range 84.4%–100%).^12^, ^17^, ^18^ However, our sensitivity of 61.0% is lower than that reported by others, whether follow‐up imaging was with NCCT only (range 95.7%–100.0%)^20^, ^24^, ^25^, ^28^ or either MRI or NCCT (range 90.0%–100.0%).^12^, ^17^, ^18^ These differences in sensitivity may be due to differences in study design. Since we only compared DECT to follow‐up with MRI, which is sensitive to detecting small hemorrhages, our study may have detected more class 1 hemorrhages than the other studies, leading to a lower reported sensitivity of hemorrhage detection for DECT.

Furthermore, it is important to note that different DECT implementations may affect performance, with differences in material discrimination reported by investigators for dual layer, twin beam, fast switching, and dual source DECT systems.^40^, ^41^, ^42^, ^43^ Almqvist et al. reported a sensitivity of 100% and specificity of 98% for fast switching kV DECT and Hu et al. reported a sensitivity of 96% and specificity of 100% for dual source DECT.^26^, ^28^ While these lacked a direct comparison with MRI, it is possible these DECT systems may perform better than the sequential DECT. While this system's is capable of projection‐based material decomposition and AEC (which was not utilized in this study), the short time interval between scans utilized in this sequential DECT system may limit temporal resolution and introduce motion‐related misregistration artifacts that could affect sensitivity for subtle hemorrhages, as reported by Li et al.^44^ These differences in acquisition and processing could impact the accuracy of material differentiation and classification.

This study has several limitations. There were no patients that were classified as class 2 hemorrhages, and few patients were classified as class 3. Additionally, 48% of class 1 cases were classified as 1B while 50% of class 3 were 3C with no 3D subtypes reflecting an uneven distribution of hemorrhage subtypes which can limit the generalizability of the findings. Future studies with larger and more diverse patient populations are needed to validate these findings and explore the potential of DECT in different clinical settings. While we included patients with follow‐up MRI within 48 h, the variability in time intervals between DECT and MRI may have influenced the detection rates, as hemorrhages could evolve between scans. Since this study only reviewed patients that received a follow‐up MRI after DECT, its findings may be biased for patients with worsening neurological conditions.

## 5 CONCLUSIONS

This study evaluated the performance of a sequential axial scanning DECT system in differentiating and grading hemorrhagic transformations in ischemic stroke patients. Our results demonstrate that DECT provides high diagnostic accuracy for clinically significant hemorrhages, while showing lower sensitivity for smaller hemorrhages that are unlikely to impact clinical management. By accurately distinguishing hemorrhage from contrast staining, DECT offers a practical and accessible alternative to MRI or serial NCCT. These findings support the integration of sequential axial scanning DECT system into stroke management workflows, enhancing diagnostic efficiency and expediting clinical decision‐making.

## AUTHOR CONTRIBUTIONS

Aroon Pressram performed observer study, analyzed data, created tables and figures for interpretation. Reordan DeJesus and Tara Massini performed image interpretation. Anna Y. Khanna assisted with explanations of clinical protocols and stroke management. Manuel Arreola supervised the research project. Izabella L. Barreto provided data, assisted with experimental design and supervised the project. All authors contributed to writing and reviewing the final manuscript.

## CONFLICTS OF INTEREST STATEMENT

I.B. has received research support from Canon Medical Systems USA for other projects.
